# Supplementary material for: Resolving mixed mechanisms of protein subdiffusion at the T cell plasma membrane
Source: Nat Commun. 2017 Jun 20;8:15851. doi: 10.1038/ncomms15851 (PMC5481838; doi:10.1038/ncomms15851)
Supplement: Supplementary Information [file ncomms15851-s1.pdf]

Type of file: pdf
Size of file: 0 KB
Title of file for HTML: Supplementary Information
Description: Supplementary Figures, Supplementary Tables.

Type of file: pdf
Size of file: 0 KB
Title of file for HTML: Peer Review File
Description:

Type of file: avi
Size of file: 0 KB
Title of file for HTML: Supplementary Movie 1
Description: Representative movie showing gp41 constructs tagged with AF594 at the plasma membrane of a live, activated Jurkat T cell as it spreads on an αCD3ε-coated coverslip that stimulates the T cell receptor.

Supplementary Figures

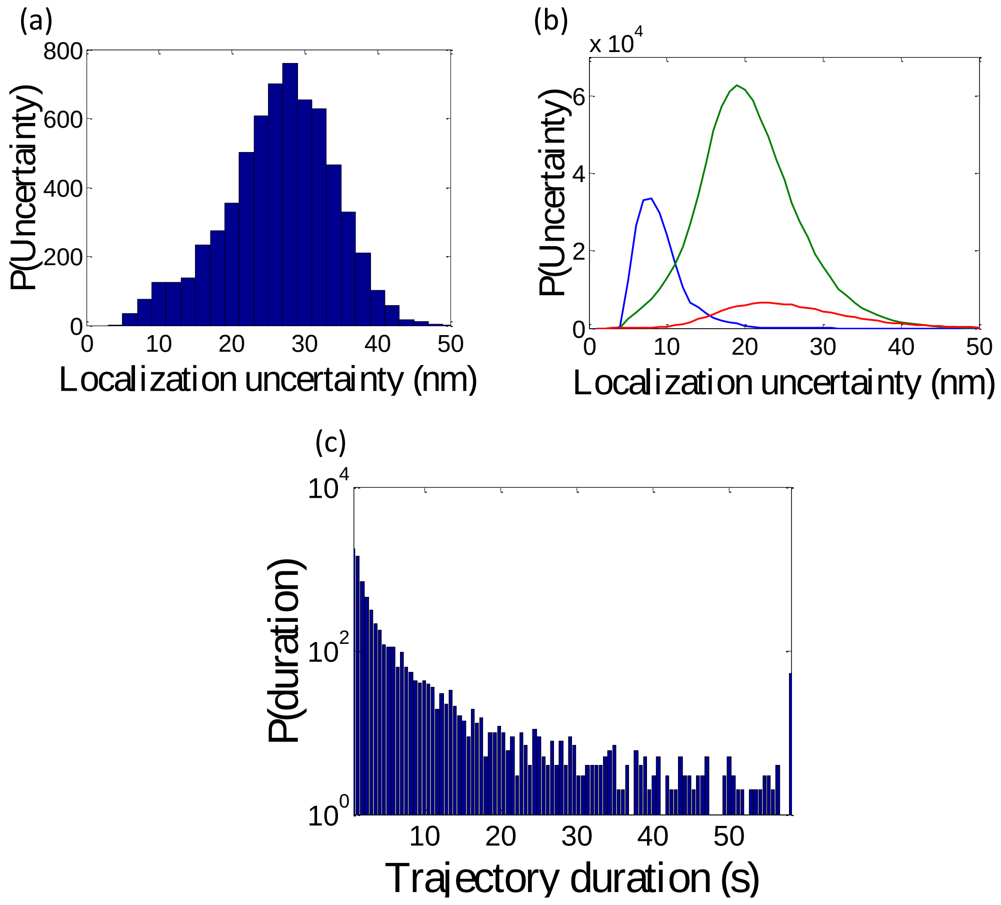

Supplementary Figure 1 – Imaging statistics

(a) Localization uncertainty of AF594 molecules. The distribution has a peak at $\sim 28\, nm$. (b) Localization uncertainty of the three subpopulations – low (blue), medium (green) and high (red) mobility states. (c) Trajectory lengths in a semi-log plot. The minimal trajectory length we consider is 50 frames which corresponds to $\sim$0.6 s.

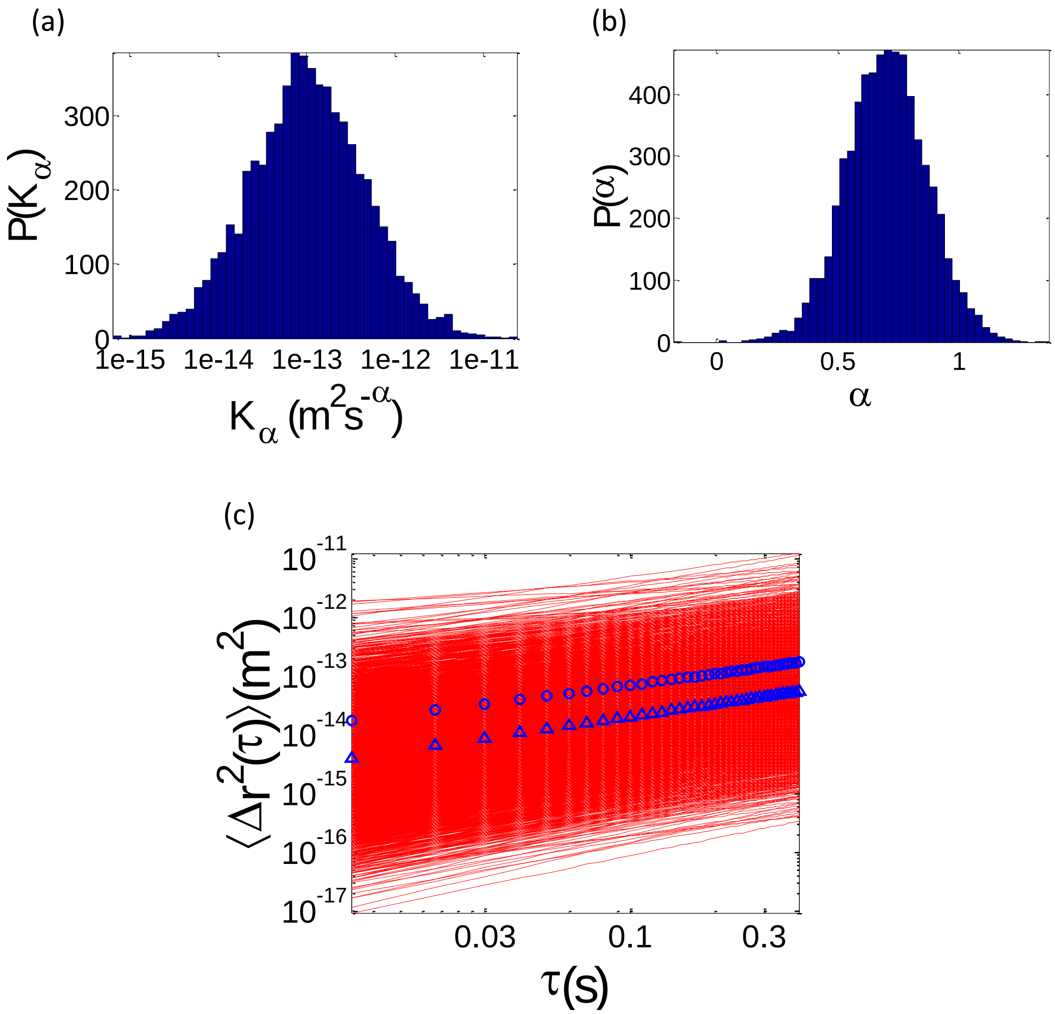

Supplementary Figure 2 – Differences between arithmetic and geometric averaging of simulated MSDs

(a,b) Distributions of (a) K_α and (b) α values of simulated mean square displacements (MSDs). K_α values are taken from a log-normal distribution with mean $3.2 \cdot 10^{-13}\, m^2 s^{-\alpha}$ and std of $8.6 \cdot 10^{-13}\, m^2 s^{-\alpha}$ and α values are taken from a normal distribution with mean 0.7 and std of 0.17. These distributions are chosen to broadly resemble the distributions of the experimental data (see (b) in the main text). (c) A log-log plot of 6400 simulated MSDs with respect to time. Shown are MSDs for single trajectories (red lines), The arithmetic mean (blue circles) and the geometric mean (blue triangles) of all simulated MSDs. The K_α values for the arithmetic and geometric means are $3.05 \cdot 10^{-13}\, m^2 s^{-\alpha}$ and $1.02 \cdot 10^{-13}\, m^2 s^{-\alpha}$ respectively. The α values for the arithmetic and geometric means are 0.65 and 0.70 respectively.

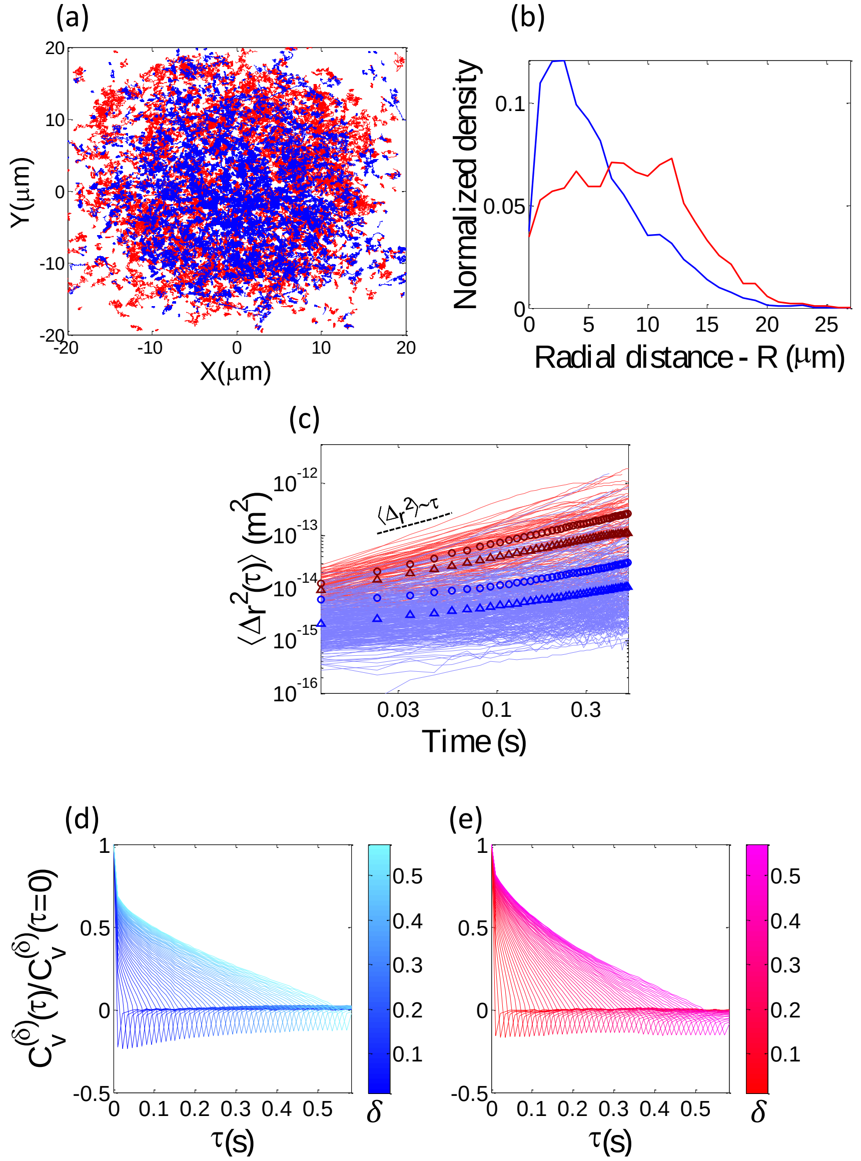

Supplementary Figure 3 - Analysis for a 2-states model

Repetition of statistical tests for segmentation done with the vbSPT method for a model of 2 mobility states. All panels are the same as the corresponding panels in Figs. 4-6. (a) Montage image of trajectories from 30 different cells. (b) Radial density distribution of mobility states. (c) A log-log plot of mean square displacements (MSDs) of the 2 different mobility subpopulations. (d)-(e) Velocity autocorrelation functions for the 2 different mobility subpopulations. Coloring is blue and red for mobility states $s_{1,2}$ in ascending order of mobility.

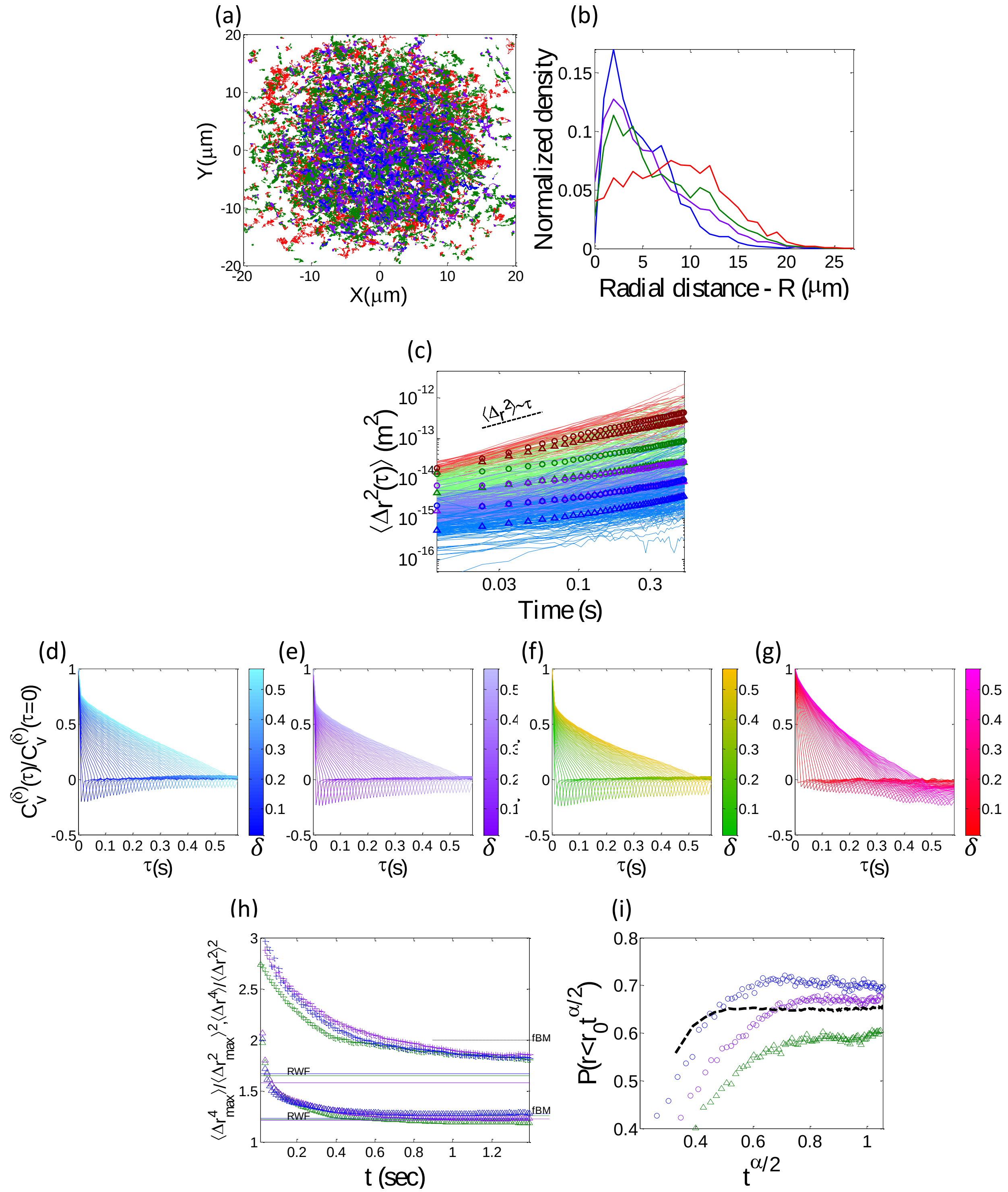

Supplementary Figure 4 – Analysis for a 4-states model

Repetition of statistical tests for segmentation done with the vbSPT method for a model of 4 mobility states. All panels are the same as the corresponding panels in Figs. 4-7. (a) Montage image of trajectories from 30 different cells. (b) Radial density distribution of mobility states. (c) A log-log plot of mean square displacements (MSDs) of the 4 different mobility subpopulations.

(d)-(g) Velocity autocorrelation functions for the 4 different mobility subpopulations. (h) Regular (MSD) moment ratios (crosses) and mean maximal excursion (MME) moment ratios (triangles). Horizontal dashed black lines mark the value 2.0 which is the expected value for the normal ratios of an fBM process and the expected value of the MME moment ratio for an fBM process. Red solid lines mark the expected values for an RWF process for the regular and MME moment ratios. (i) Growing sphere analysis. Coloring is blue, purple, green and red for mobility states $s_{1,2,3,4}$ in ascending order of mobility.

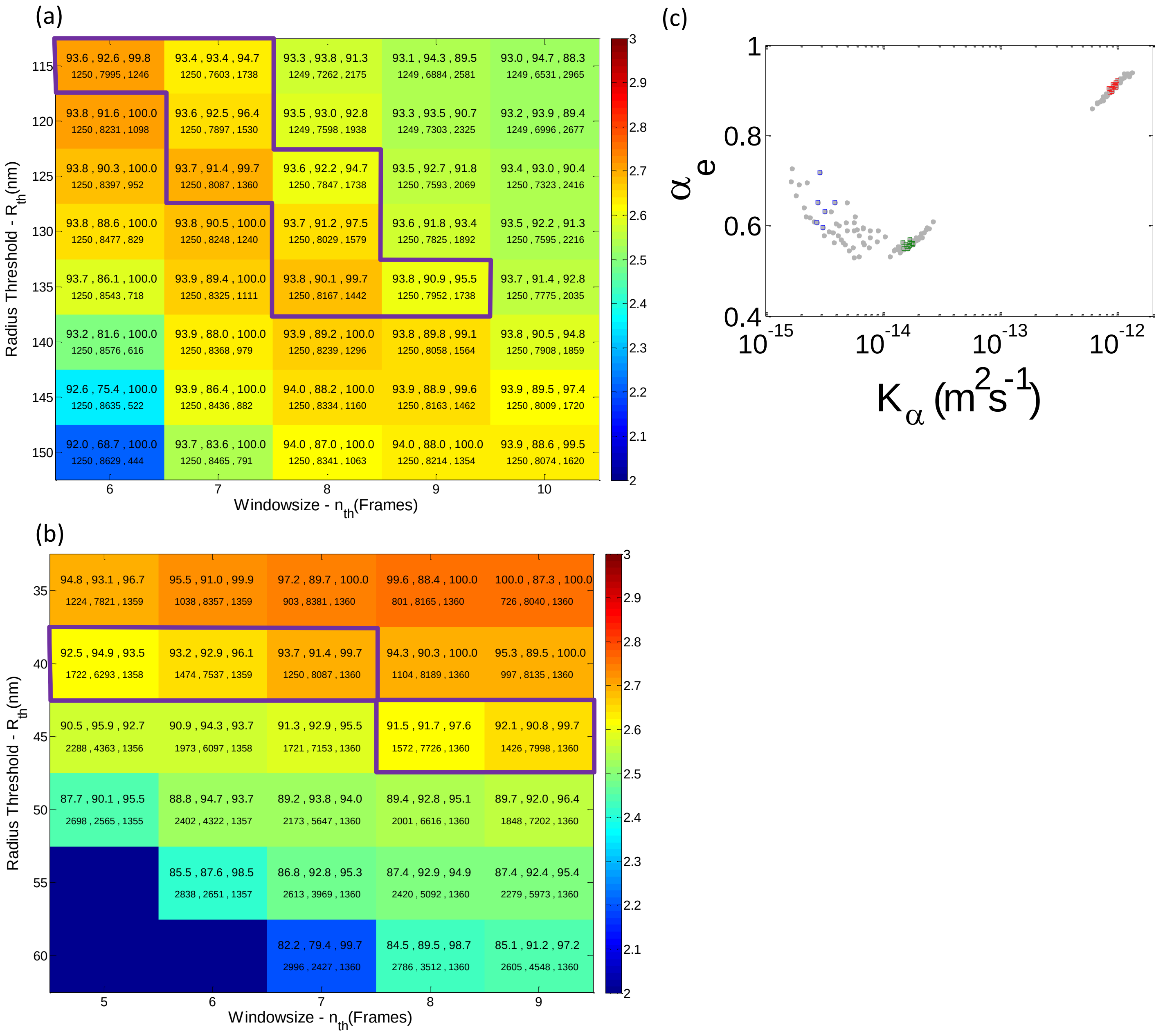

Supplementary Figure 5 – Guidelines for choice of threshold set for CER segmentation method

(a,b) Heatmaps of threshold set scores. (a) Fixed thresholds $\{R_{th}^{s_1,s_2} = 40\ nm, n_{th}^{s_1,s_2} = 7\ \text{frames}\}$ and variable thresholds $\{R_{th}^{s_2,s_3} \in (115,150)\ nm, n_{th}^{s_2,s_3} \in (6,10)\ \text{frames}\}$. (b) Variable thresholds $\{R_{th}^{s_1,s_2} \in (35,60\ nm), n_{th}^{s_1,s_2} \in (5,9)\ \text{frames}\}$ and fixed thresholds $\{R_{th}^{s_2,s_3} = 125\ nm, n_{th}^{s_2,s_3} = 7\ \text{frames}\}$. Values in each location are the on-diagonal values $a_{i=j}^{j}$ and the number of trajectory segments in each subpopulation in the following order: $\begin{bmatrix} a_1^1 & a_2^2 & a_3^3 \\ N_1 & N_2 & N_3 \end{bmatrix}$. Coloring is according to the total score $\sum_{i=j}\left(a_i^j\right)^2 - \sum_{i\neq j}\left(a_i^j\right)^2$ which has an upper bound at 3.0. Threshold sets marked

in a purple outline are ones which are considered preferable according to the guidelines found in the SI. (c) Values of α_e vs $K_{\alpha,e}$ for a representative range of threshold sets. Grey points are values for all threshold sets and squares are the values for threshold sets preferable according to the guidelines. Colors are for low (blue), medium (green) and high (red) mobility states.

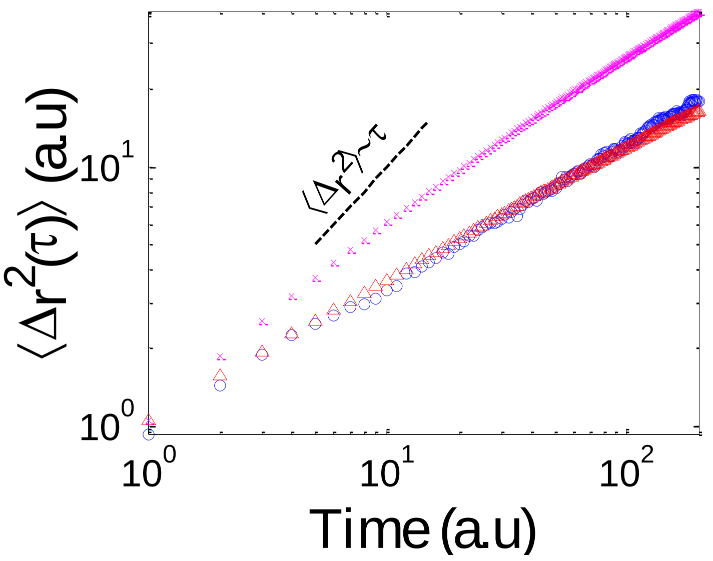

Supplementary Figure 6 – Confined particles simulation

A log-log plot of mean square displacements (MSDs) of confined particles undergoing an fBM process. Blue circles, red triangles and magenta crosses are ensemble, time and MME averages respectively. 500 particles were simulated for a duration of 500 time-steps. All particles have an α value of 0.5. The region of confinement is a circle with a radius of 10 units. The particle mean step-size is ~0.9 units. The resulting α values are $\alpha_e = 0.56 \pm 0.01$; $\alpha_t = 0.50 \pm 0.01$; $\alpha_{MME} = 0.63 \pm 0.02$.

Supplementary Tables

Supplementary Table 1 - Subdiffusive power α and $K_\alpha[m^2 s^{-\alpha}]$ for the 3 mobility states found by the vbSPT method

	State 1	State 2	State 3
α_e	$0.61(9)$	$0.63(5)$	$0.81(2)$
α_t	$0.63(7)$	$0.50(5)$	$0.72(1)$
α_{MME}	$0.66(3)$	$0.58(6)$	$0.84(1)$
$K_{\alpha,e}$	$2.0(2)\cdot 10^{-14}$	$8.0(5)\cdot 10^{-14}$	$6.3(1)\cdot 10^{-13}$
$K_{\alpha,t}$	$0.8(1)\cdot 10^{-14}$	$2.5(1)\cdot 10^{-14}$	$3.4(1)\cdot 10^{-13}$
$K_{\alpha,MME}$	$2.0(1)\cdot 10^{-14}$	$6.6(5)\cdot 10^{-14}$	$6.7(1)\cdot 10^{-13}$

Comments:
The ensemble, time average and MME values are calculated from the fit to each MSD while taking into account weights according to the SEM error of each data point in the MSD curve. The error of the last digit is shown in parenthesis. The error is estimated by the standard deviation of fitted values over multiple time windows of the MSD.

Supplementary Table 2 - Subdiffusive power α and $K_\alpha[m^2 s^{-\alpha}]$ for the 4 mobility states found by the vbSPT method

	State 1	State 2	State 3	State 4
α_e	$0.63(8)$	$0.58(7)$	$0.67(5)$	$0.81(2)$
α_t	$0.68(7)$	$0.57(5)$	$0.50(9)$	$0.74(3)$
α_{MME}	$0.68(2)$	$0.62(1)$	$0.59(9)$	$0.86(2)$
$K_{\alpha,e}$	$1.3(1)\cdot 10^{-14}$	$3.8(3)\cdot 10^{-14}$	$1.3(1)\cdot 10^{-13}$	$7.7(2)\cdot 10^{-13}$
$K_{\alpha,t}$	$0.6(1)\cdot 10^{-14}$	$1.2(1)\cdot 10^{-14}$	$0.38(3)\cdot 10^{-13}$	$4.6(2)\cdot 10^{-13}$
$K_{\alpha,MME}$	$1.27(4)\cdot 10^{-15}$	$3.22(3)\cdot 10^{-14}$	$1.0(1)\cdot 10^{-13}$	$9.0(3)\cdot 10^{-13}$

Comments:
The ensemble, time average and MME values are calculated from the fit to each MSD, while taking into account weights according to the SEM error of each data point in the MSD curve Supplementary Figure 4(c). The error of the last digit is shown in parenthesis.
